# Prevalence and Risk Factors of Clostridium difficile Infection Among Patients Hospitalized for a Flare of Inflammatory Bowel Disease in King Abdulaziz University Hospital

**DOI:** 10.7759/cureus.48451

**Published:** 2023-11-07

**Authors:** Areej A Samman, Mona Alfares, Raghad A Bajabur, Roaa O Alnefaie, Lama S Alwadai, Weaam W Murad, Bayan M Dahal

**Affiliations:** 1 Internal Medicine, King Abdulaziz University Faculty of Medicine, Jeddah, SAU; 2 Infectious Diseases, King Abdulaziz University Hospital, Jeddah, SAU

**Keywords:** (ibd) inflammatory bowel disease, extraintestinal manifestations in inflammatory bowel disease, community-acquired clostridium difficile infection (cdi), complications of inflammatory bowel disease, recurrent clostridium difficile infection

## Abstract

Background

The gram-negative anaerobe Clostridium difficile is the main infectious cause of pseudomembranous colitis and infectious diarrhea in hospitalized patients. Inflammatory bowel disease (IBD) patients have been proven to have higher rates of Clostridium difficile infection (CDI). Antibiotic use is the most well-known of the several risk factors for CDI. A few more are advanced age, previous hospitalization, increased severity of an underlying illness, gastrointestinal surgery, and proton pump inhibitors. This study aimed to find out which factors predict CDI in IBD patients at King Abdulaziz University Hospital in Jeddah.

Methods

We conducted a retrospective cohort study of all inflammatory bowel disease patients who developed CDI with a total sample of 602 patients from 2009 through 2022 at King Abdulaziz University in Jeddah, Saudi Arabia. We identified the clinical data of patients diagnosed with CDI and admitted to the hospital for either diagnosis or follow-up, and we measured the frequencies and percentages as qualitative data and the mean ( standard deviation) as quantitative variables. A chi-square test was used to estimate the correlation between Clostridium difficile infections and multiple factors, including a history of previous hospitalizations, recent flares, intestinal manifestations, extraintestinal manifestations, comorbidities, and IBD medications. Meanwhile, independent t-tests were performed to analyze the continuous variables.

Results

Out of 602 IBD patients, 53 patients (8.8%) had a confirmed CDI test using an immunoassay for Clostridium difficile toxins A and B. Most of the patients were female and nonsmokers. Regarding colonic involvement, 47 individuals with the disease extending to their large colon also evaluated positive for CDI. Among patients with a positive history of CDI, there were 21 patients with a recent flare-up of fewer than five episodes, five patients had more than five episodes, and the rest did not have any recent flare-ups. Also, IBD patients were significantly at a higher risk for intestinal resection.

Conclusion

IBD patients are more susceptible to CDI due to flare-ups that require hospitalization and their medications. As a result, clinicians must consider CDI testing in IBD patients who are hospitalized and who are receiving medication to ensure early diagnosis and therapy.

## Introduction

Clostridium difficile infection (CDI) is a severe infection that results in significant mortality and morbidity worldwide [1]. Clostridium difficile (CD) is a gram-positive, anaerobic, and spore-forming bacterium that produces toxins that destroy the epithelium of the intestine [2,3]. The clinical manifestation of CDI is individual-dependent and ranges from asymptomatic carriers to life-threatening colitis [4]. However, the most common clinical picture is watery diarrhea, along with fever, abdominal pain, vomiting, nausea, loss of appetite, and weakness [2]. Over the past few years, there has been a significant rise in CDI incidence recorded globally [4]. Hospitalized patients of older age (more than 65 years) with exposure to antibiotics such as aminoglycosides, lincomycin, tetracyclines, erythromycin, clindamycin, penicillins, cephalosporins, and fluoroquinolones, which are commonly used in the treatment of bacterial infections in clinical settings, are at the highest risk for developing CDI [2]. The use of immunosuppressants, chronic kidney diseases, transplantations, immunological incompetence induced by malignant neoplasms, and gastrointestinal surgeries are other well-known risk factors [5,6]. Inflammatory bowel disease (IBD) patients have been proven to have greater CDI rates and significantly higher morbidity and mortality compared to CDI patients without IBD [7]. According to the treatment, the most effective antibiotic for CDI is vancomycin; in addition, metronidazole and clindamycin are other effective alternatives. In complicated cases, surgical removal of the colon may be the only life-saving choice [8]. IBD is a progressive and chronic condition characterized by inflammation of the gastrointestinal tract [3]. It can be classified into two major forms: Crohn’s disease (CD) and ulcerative colitis (UC) [9]. The pathophysiology of IBD, which includes the distribution of gut microbiota composition, plays an important role in the development of CD infection [3].

Environmental and genetic factors played a role in the development of the disease. Cigarette smoking and chromosomes 12 and 16 are the main predisposing factors [10]. The correlation between CDI and IBD, a retrospective study in the United States (US) with 357,242 IBD participants, revealed an increase in the incidence of CDI, specifically in ulcerative colitis patients [11]. Another retrospective cohort study has been done in the US for all IBD patients with bacterial gastrointestinal infections, specifically CD, and claimed that the case fatality and prevalence of IBD patients with CDI have markedly increased, particularly for those patients with ulcerative colitis [12]. In 2017, a retrospective study showed that IBD patients had a 6.7% incidence of CDI, and it was determined that patients with IBD are more prone to CDI at younger ages and frequently lack typical risk factors [4].

There have been no previous studies determining the incidence and outcomes of CDI among hospitalized IBD patients in Saudi Arabia. As a consequence of the limited research on this topic, we aimed to determine the predictors of CDI in IBD patients from 2004-2022 at King Abdulaziz University Hospital in Jeddah, Saudi Arabia.

## Materials and methods

An analytical retrospective record review was done to allow multiple exposures to be studied, thus identifying the effects of each exposure on the disease. Data were obtained from the medical department at the King Abdulaziz University Hospital in Jeddah, Saudi Arabia. The sample size targeted all the patients who were diagnosed with IBD from 2004 to 2022, which is 710 patients.

The inclusion criteria are defined as patients who are already diagnosed with IBD and are re-admitted through the ER or had multiple visits to the OPD, needed to change their medications, or underwent IBD-related emergency surgeries. IBD patients with CDI who were hospitalized due to flares met the inclusion criteria. However, patients under the age of 18 who were diagnosed outside of KAUH were excluded.

Ethical approval was obtained from the Unit of Biomedical Ethics at KAUH (reference number: 24821).

Demographic and other data were collected through Google Forum Sheets with the following variables: nationality, gender, age, height, weight, BMI, type of IBD (UC) or CD), duration of diseases since diagnosis, colonic involvement, smoking status, history of CDI, history of previous hospitalization (for <7 days or >7 days), previous flare-up episodes within the last two years, gastrointestinal manifestations (intestinal resection, primary sclerosing cholangitis, hepatitis, hepatic abscess, portal vein thrombosis, hepatic steatosis, pancreatitis, primary biliary cirrhosis, cholelithiasis, autoimmune pancreatitis), extra-intestinal manifestation (osteoporosis, osteonecrosis, osteomyelitis, large airway disease, pneumonia, conjunctivitis, episcleritis, enterocutaneous fistula, pyoderma gangrenosum, erythema nodosum), comorbidities (hypertension (HTN), diabetes mellitus (DM), ischemic heart disease, chronic heart failure, autoimmune disease, chronic kidney disease, cerebrovascular disease, thyroid disease) and IBD medications (corticosteroid, immunosuppressant, biological agent).

Then, the data was entered through Microsoft Excel 2016 (Microsoft Corporation, Redmond, WA). Data analysis was performed using the Statistical Package for Social Sciences (SPSS) version 21 (IBM Corp., Armonk, NY). Qualitative data were presented as frequencies and percentages, and quantitative variables were represented as the mean ( standard deviation). Independent t-tests were used to compare continuous variables, and the chi-square test was used to compare categorical variables. Statistical significance was defined as a p-value below 0.05.

## Results

Baseline characteristics of patients 

In this study, we included 602 patients who were diagnosed with IBD where 39% of them were diagnosed with UC and 61% with CD. Moreover, the females are relatively higher than males with a mean age of 36.58 years old (SD=14.03). We conducted our study on 24 different nationalities, and the majority were Saudis (73.8%). Moreover, colonic involvements were reported in 73.8% of the patients, 95.7% were non-smokers, and 41.5% had normal weight. In addition, 57.1% of the patients with IBD were not hospitalized for symptoms (Table 1).

**Table 1:** Baseline characteristics of the patients

		Count	Column N %
Age		36.58 (14.03)	
Nationality	Saudi	444	73.8%
	Non-Saudi	158	26.2%
Gender	Male	294	48.8%
	Female	308	51.2%
BMI	Mean (SD)	24.58 (6.14)	
BMI	Underweight	85	15.6%
	Normal weight	226	41.5%
	Overweight	141	25.9%
	Obese	92	16.9%
Type of IBD	Crohn’s disease (CD)	367	61.0%
	Ulcerative colitis (UC)	235	39.0%
Colonic involvement	No	158	26.2%
	Yes	444	73.8%
Smoking status	Non-smoker	576	95.7%
	Smoker	15	2.5%
	Ex-smoker	11	1.8%
History of Clostridium difficile infection	No	549	91.2%
	Yes	53	8.8%
History of previous hospitalizations	No	344	57.1%
	Yes, for less than 7 days	109	18.1%
	Yes, for more than 7 days	149	24.8%

In addition, 53 patients (8.8%) of all patients had a confirmed CDI test using an immunoassay for Clostridium difficile toxins A and B where 29 patients were females while 24 were males with no significant difference between the two genders in the prevalence of confirmed CDI (9.4% of females compared with 8.2% of male, P=0.588). Moreover, 54.7% of the patients with CDI were hospitalized for more than seven days while 24.5% were hospitalized for less than seven days, and 20.8% of them did not stay at the hospital after their diagnosis but were discharged on medication. Although the majority of our population is non-smokers, we did notice that 15 of them are smokers but didn’t have a confirmed history of CDI. Regarding colonic involvement, 47 of the individuals who had the disease extended to their large colon also tested positive for CDI. Among patients with a positive history of CDI, there were 21 patients with a recent flare-up of less than five episodes, five patients had more than five episodes, and the rest didn’t have any recent flare-ups with a significant difference in the rate of flare-ups between patients with and without CDI with more frequency in patients with CDI (P=0.000) (Table 2).

**Table 2:** The relation between demographic factors and confirmed CDI *Marked values indicate a significant p-value lower than 0.5 CDI: Clostridium difficile infection

		History of Clostridium difficile infection				
		No		Yes		p-value
		Count	Column N %	Count	Column N %	
Gender	Male	270	49.2%	24	45.3%	0.588
	Female	279	50.8%	29	54.7%	
Colonic involvement	No	151	27.5%	7	13.2%	0.024*
	Yes	398	72.5%	46	86.8%	
Smoking status	Non-smoker	525	95.6%	51	96.2%	0.265
	Smoker	15	2.7%	0	0.0%	
	EX smoker	9	1.6%	2	3.8%	
History of previous hospitalizations	No	333	60.7%	11	20.8%	0.000*
	Yes, for less than 7 days	96	17.5%	13	24.5%	
	Yes, for more than 7 days	120	21.9%	29	54.7%	
Any previous flare-up episodes in the last 2 years?	No	458	83.4%	27	50.9%	0.000*
	Yes, less than 5 episodes	79	14.4%	21	39.6%	
	Yes, more than 5 episodes	12	2.2%	5	9.4%	

Gastrointestinal tract (GIT) manifestation related to CDI

**Figure 1:**
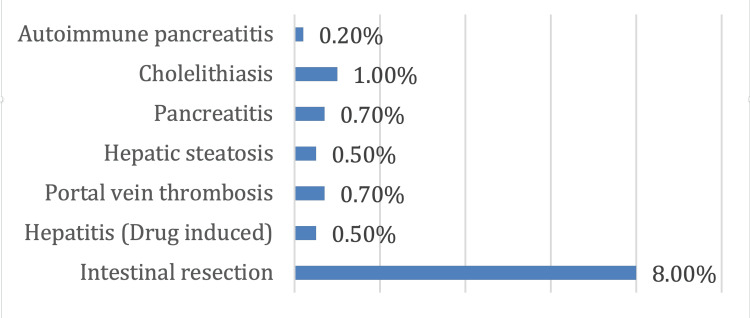
GIT manifestation related to CDI GIT: gastrointestinal tract; CDI: Clostridium difficile infection

Intestinal resection was conducted in 8% of the patients followed by cholelithiasis, as reported in 1% of cases, and portal vein thrombosis in 0.7% (Figure 1).

We found a strong and significant relationship between the type of IBD and intestinal resection (p-value = 0.001). Crohn’s patients were more susceptible to intestinal resection (CD 85.4%, UC 14.6%). Patients who underwent intestinal resection seemed to have a significant relationship with a history of having CDI (p-value = 0.001). IBD patients who have a previous history of Clostridium difficile infection are more susceptible to intestinal resection (22.9%). Regarding our results, we found that IBD patients who have been hospitalized are at higher risk for intestinal resection (for more than 7 days, 54.2%; less than 7 days, 37.5%). While other patients developed portal vein thrombosis (for more than 7 days, 23.4%; less than 7 days, 18.1%) (p-value 0.029) (Table 3). Moreover, there is a positive relationship between the duration of the disease and intestinal resection in IBD patients (p-value = 0.002), as well as between the duration of the disease and portal vein thrombosis (p-value = 0.004) and with cholelithiasis (p-value = 0.033). There is a relationship between age and portal vein thrombosis (p-value = 0.006) and between age and cholelithiasis (p-value = 0.047). Regarding recent flare-up history, patients who had less than five episodes of flare-ups were 27.1%, and patients who had more than five episodes were 16.7%. However, we noticed a significant relationship: patients who had more than five episodes also had portal vein thrombosis (p-value = 0.006). We found that nationality may also play a role in IBD patients, making them at higher risk for surgical procedures such as intestinal resection (p-value = 0.037).

**Table 3:** Association between demographic factors and intestinal resection *Marked values indicate a significant p-value of lower than 0.5

		Intestinal resection				
		No		Yes		P-value
		Count	Column N %	Count	Column N %	
Nationality	Saudi	416	75.1%	28	58.3%	0.011*
	Non-Saudi	138	24.9%	20	41.7%	
Gender	Male	273	49.3%	21	43.8%	0.462
	Female	281	50.7%	27	56.3%	
Type of IBD	Crohn’s disease	326	58.8%	41	85.4%	0.000*
	Ulcerative colitis	228	41.2%	7	14.6%	
Colonic involvement	No	153	27.6%	5	10.4%	0.009*
	Yes	401	72.4%	43	89.6%	
Smoking status	Non-smoker	531	95.8%	45	93.8%	0.731
	Smoker	13	2.3%	2	4.2%	
	EX smoker	10	1.8%	1	2.1%	
History of Clostridium difficile infection	No	512	92.4%	37	77.1%	0.000*
	Yes	42	7.6%	11	22.9%	
History of previous hospitalization	No	340	61.4%	4	8.3%	0.000*
	Yes, for less than 7 days	91	16.4%	18	37.5%	
	Yes, for more than 7 days	123	22.2%	26	54.2%	

Extra-GIT manifestation related to CDI

Regarding the extra-intestinal manifestation, surprisingly, we discovered that IBD patients with a history of CDI did not significantly differ from those without CDI in terms of their risk of developing extra-intestinal manifestation. Furthermore, our findings demonstrated a significant association between CDI and enterocutaneous fistula in patients with IBD (p-value = 0.009) (Table 4). Even though osteoporosis and CDI were not significantly correlated, it was unexpected that osteoporosis was statistically related to other multiple factors, including recent flare-ups (P = 0.001) and disease duration (P = 0.003). weight (P = 0.033), height (P = 0.008). Additionally, it was considerably more frequent to see a positive relationship between a previous hospitalization history or a recent flare-up history associated with other extra-intestinal manifestations. Moreover, experiencing hypertrophic osteoarthropathy is significantly associated with a previous hospitalization history (p-value = 0.011) and a recent history of flare-ups (P = 0.014). While experiencing osteonecrosis, conjunctivitis and enterocutaneous fistula were only correlated with a previous history of hospitalization with p-values of 0.02, 0.038, and 0.003, respectively. According to our findings, there is an increased likelihood of acquiring extra-intestinal manifestations with age. This showed a significant relationship with pneumonia (P = 0.040), congestive heart disease (P = 0.00), and episcleritis (P = 0.048). Nevertheless, there was no significant relationship between gender, type of IBD, colonic involvement, BMI, and nationality and extra-intestinal manifestations.

**Table 4:** The association between CDI and incidence of extra-gIT manifestation related to CDI *Marked values indicate a significant p-value lower than 0.5 GIT: gastrointestinal tract; CDI: Clostridium difficile infection

		History of Clostridium difficile infection				
		No		Yes		P-value
		Count	Column N %	Count	Column N %	
Osteoporosis	No	525	95.6%	50	94.3%	0.665
	Yes	24	4.4%	3	5.7%	
Osteonecrosis	No	544	99.1%	52	98.1%	0.495
	Yes	5	0.9%	1	1.9%	
Osteomyelitis or bone abscess	No	545	99.3%	53	100.0%	0.533
	Yes	4	0.7%	0	0.0%	
Hypertrophic osteoarthropathy	No	543	98.9%	51	96.2%	0.104
	Yes	6	1.1%	2	3.8%	
Large airway disease	No	542	98.7%	52	98.1%	0.710
	Yes	7	1.3%	1	1.9%	
Pneumonia	No	542	98.7%	53	100.0%	0.408
	Yes	7	1.3%	0	0.0%	
Congestive heart disease	No	544	99.1%	53	100.0%	0.485
	Yes	5	0.9%	0	0.0%	
Conjunctivitis	No	538	98.0%	53	100.0%	0.298
	Yes	11	2.0%	0	0.0%	
Episcleritis	No	545	99.3%	53	100.0%	0.533
	Yes	4	0.7%	0	0.0%	
Enterocutaneous fistula	No	543	98.9%	50	94.3%	0.009*
	Yes	6	1.1%	3	5.7%	
Pyoderma gangrenous	No	545	99.3%	53	100.0%	0.533
	Yes	4	0.7%	0	0.0%	
Erythema nodosum	No	548	99.8%	53	100.0%	0.756
	Yes	1	0.2%	0	0.0%	

Comorbidity related to CDI

There was a significant relationship between certain comorbidities and the type of IBD a patient had. Significant comorbidities related to the type of IBD include HTN (p-value = 0.00), UC being the most common in hypertensive patients, with 3.8% of them having UC compared to the 1.5% of hypertensive patients who have CD, congestive heart failure (CHF) (p-value = 0.012), and thyroid diseases (p-value = 0.009). Meanwhile, an insignificant relationship has been drawn between the type of IBD a patient has and the following comorbidities: ischemic heart disease (IHD) (p-value = 0.160), DM (p-value = 0.52), chronic kidney disease (CKD) (p-value = 0.163), cerebrovascular diseases (p-value = 0.773), and autoimmune diseases (p-value = 0.671) (Table 5). There has been no significant relationship established between smoking patients and each comorbidity a patient has. Colonic involvement is significantly relevant to HTN (p-value = 0.017) and DM (p-value = 0.026). As for the rest of the comorbidities, which are IHD, autoimmune diseases, CKD, cerebrovascular diseases, and thyroid diseases, an insignificant relationship ties them to smoking. Comorbidities are significantly related to the duration of the diseases. The significant comorbidities are HTN (p-value = 0.001), DM (p-value = 0.041), CHF (p-value = 0.003), autoimmune diseases (p-value = 0.043), and CKD (p-value = 0.040) while the insignificant ones are cerebrovascular diseases, IHD, and thyroid diseases. There has been no significant relationship found between CDI and any of the following comorbidities: hyperparathyroidism (HPT), DM, CHF, IHD, CKD, autoimmune diseases, cerebrovascular diseases, and thyroid diseases. In addition to a recent history of CDI, a recent history of flare-ups is insignificantly correlated to the following comorbidities: HPT, DM, IHD, CHF, CKD, and thyroid diseases. The only exception is autoimmune diseases (p-value = 0.008) and cerebrovascular diseases (p-value = 0.018), where a significant relationship has been found linking the two comorbidities to a recent history of flare-ups. A significant correlation between previous hospitalization and DM (p-value = 0.011), IHD (p-value 0.038), CHF (p-value 0.019), autoimmune diseases (p-value = 0.005), cerebrovascular diseases (p-value = 0.006), as well as thyroid diseases (p-value = 0.044), has been established while there is no significant relation between both HPT and CKD and the history of previous hospitalization.

**Table 5:** The relation between the type of IBD and comorbidity *Marked values indicate a significant p-value lower than 0.5 IBD: inflammatory bowel disease

		Type of IBD				
		Crohn’s disease		Ulcerative colitis		P-value
		Count	Column N %	Count	Column N %	
Hypertension	No	358	97.5%	212	90.2%	0.000*
	Yes	9	2.5%	23	9.8%	
Diabetes mellitus	No	347	94.6%	212	90.2%	0.52*
	Yes	20	5.4%	23	9.8%	
Ischemic heart disease	No	361	98.4%	227	96.6%	0.160
	Yes	6	1.6%	8	3.4%	
Chronic heart failure	No	367	100.0%	231	98.3%	0.012*
	Yes	0	0.0%	4	1.7%	
Autoimmune disease	No	351	95.6%	223	94.9%	0.671
	Yes	16	4.4%	12	5.1%	
Chronic kidney disease	No	365	99.5%	231	98.3%	0.163
	Yes	2	0.5%	4	1.7%	
Cardiovascular disease	No	363	98.9%	233	99.1%	0.773
	Yes	4	1.1%	2	0.9%	
Thyroid disease	No	358	97.5%	219	93.2%	0.009
	Yes	9	2.5%	16	6.8%	

Drugs related to CDI

Regarding the IBD medications as a risk factor that predisposes patients to CDI, we found that there is a significant relationship between the type of IBD and the use of corticosteroids (p-value = 0.018). Compared to UC patients, CD patients appear to receive corticosteroids more commonly (64.7% CD, 35.3% UC). Also, we have seen a relationship between the type of IBD and any other immunosuppressant medications (p-value = 0.002). As mentioned above, Crohn’s patients seem to receive more immunosuppressive medications than UC patients (69.9% CD, 30.1% UC). There is also a significant relationship between the use of infliximab and the type of IBD (p-value = 0.000). Patients with Crohn's disease who use infliximab are counted at 76.2%, compared to UC patients (23.8%). Interestingly, we found that PPI and antibiotics also had a relationship with the type of IBD (p-values for both = 0.000); however, we found that the use of NSAID did not play a significant role in the type of IBD (Table 6). The use of corticosteroids and other immunosuppressive medications was significantly associated with patients whose anatomical distribution involved the colon (p-values = 0.014 and 0.007, respectively). Patients with a history of CDI and those on any kind of IBD medication have been proven to be significantly correlated. Furthermore, a significant relationship is documented between IBD medications and previous hospitalization for more than seven days (p-value = 0.000). Additionally, the length of the disease appears to be important in relation to corticosteroids (p-value = 0.000), immunosuppressants (P = 0.000), infliximab (P = 0.019), and antibiotics (P = 0.005). Moreover, patients receiving corticosteroids, infliximab, PPI, and antibiotics have been proven to develop more IBD flare-ups in the last two years with a p-value of 0.000 compared to other medications such as immunosuppressants and non-steroidal anti-inflammatory drugs (NSAIDs).

**Table 6:** The relation between the type of IBD and the drug related to CDI *Marked values indicate a significant p-value lower than 0.5 PPI: proton pump inhibitors; NSAIDs: non-steroidal anti-inflammatory drugs

		Type of IBD				
		Crohn’s disease		Ulcerative colitis		P-value
		Count	Column N %	Count	Column N %	
Corticosteroids	No	120	32.7%	100	42.6%	0.014*
	Yes	247	67.3%	135	57.4%	
Immunosuppressant	No	223	60.8%	173	73.6%	0.001*
	Yes	144	39.2%	62	26.4%	
Anti-TNFa (infliximab)	No	146	39.8%	166	70.6%	0.000*
	Yes	221	60.2%	69	29.4%	
PPI	No	137	37.3%	138	58.7%	0.000*
	Yes	230	62.7%	97	41.3%	
Antibiotics	No	123	33.5%	119	50.6%	0.000*
	Yes	244	66.5%	116	49.4%	
NSAIDs	No	218	59.4%	138	58.7%	0.869
	Yes	149	40.6%	97	41.3%	

## Discussion

Our study analyzed a large group (n = 602) of IBD patients for epidemiologic and comorbid conditions. Of these IBD patients, 53 (8.8%) had proven CDI. This rate of incidence is relatively similar to other studies of CDI in IBD with 3%-6% rates [4,11,13,14]. The CDI patients in our study were diagnosed using an immunoassay for toxins A and B. The mean age for patients with IBD who contracted CDI was only 36.5 years. This is in stark contrast to the mean age of non-IBD patients most susceptible to CDI, which ranges from 65 to 75 years of age depending on the study [15-18]. Increased age is a well-recognized risk factor for CDI; however, some studies have shown that IBD patients appear to be more susceptible at a younger age [12,13,19,20]. A recent assessment of 42 studies conflicts with our findings: the incidence of CDI was higher in patients with CD than in those with UC, and it was higher in patients hospitalized for a flare of the disease and in those who were asymptomatic [21]. Our study shows that CDI is frequently associated with flare-ups of IBD that require hospitalization. This justifies the need for testing patients with IBD for CDI upon admission to the hospital; 30 of the CDI patients were in the hospital for longer than 7 days while just 13 were there for a shorter duration. A significant relationship has been found between hospitalization and the following comorbidities: HTN, DM, IHD, autoimmune diseases, and thyroid diseases. Therefore, it can be concluded that a patient with HTN is more likely to be hospitalized and thus more likely to test positive for CDI. A study conducted in India found that the most common cause of hospitalization in adult patients aged 40 or more is noncommunicable diseases (NCDs) like HTN and DM [22]. As a result, it would be advised that comorbidities be controlled in the best way possible so as to avoid consequential hospitalization and a higher chance of CDI. Moreover, the occurrence of CDI appears to be related positively to flare-ups and this may be explained by the ability of both systemic and gastrointestinal infections to trigger the already excited gastrointestinal mucosal system in IBD due to immune activation [12]. According to our findings, IBD patients who have been hospitalized are at higher risk for intestinal resection. In fact, a previous study reported that individuals with IBD who also had related C. difficile had higher rates of colectomy and four-fold higher mortality than those with no underlying IBD [23]. In addition, according to our study, a relationship between colonic involvement and both HTN and DM has been proven. This highlights the urgency of controlling HTN and DM in patients with either or both of them. Patients with at least one CDI were more likely to have extraintestinal manifestations of IBD than those patients without CDI (43% versus 28%) [4]. In addition, our study showed a strong relationship between the occurrence of CDI and extraintestinal manifestations of IBD (arthritis, osteoporosis, osteocalcin, pyoderma gangrenosum, psoriasis, and chronic pancreatitis), we found that IBD patients with at least one CDI were more likely to develop Enterocutaneous fistula than those without CDI (5.7% versus 1.1%). In regard to IBD medications, patients on any type of IBD medication are statistically associated with a history of CDI. This may be due to the immunosuppressive effects of these agents [4,12]. This emphasizes that continued monitoring of these patients is required to ensure the early diagnosis of CDI. Moreover, it is documented in our study that Crohn's individuals receive more corticosteroids, immunosuppressants, and infliximab, which may predispose them to CDI, although the prevalence of C. difficile among those with UC and those with CD is not demonstrated in this study. We found that recent use of NSAIDs was an independent predictor for CDI. In accordance with our results, some societies have expressed that the evidence of the destructive effects of NSAIDs was clear enough to make patients with IBD avoid these medications. Antibiotics were used by 90.6% of the patients who had suffered from CDI along with IBD. In contrast, only 43.2% of patients don’t develop CDI while exposed to antibiotics. The antibiotics are believed to suppress normal colonic bacteria that usually keep Clostridium difficile from multiplying and causing diarrhea and inflammation. We are reporting approximately a two-fold increased exposure to antibiotics among individuals with IBD who are developing CDI. Previous flare-ups more than five times in the last two years were higher in patients who were exposed to antibiotics compared to patients who were not on antibiotics (83.3% versus 16.7%). Our study showed that colonic involvement was independent of antibiotic use. To corroborate, a study that was done in Canada reported higher rates of CDI among patients receiving vancomycin [24]. It is known that immunosuppressant agents increase the susceptibility to opportunistic infections; in our study, the use of immunosuppressants did not appear to give a predictable risk for CDI in IBD patients.

Limitations

This study was retrospective. We were unable to evaluate the severity of CDI due to the large proportion of community-acquired and indeterminate infections. Data were collected from the hospital system. Similarly, our population consists of only those patients who were admitted to our hospital with CDI or were followed up by one of our outpatient providers around the time of diagnosis. There is a likelihood that IBD patients were diagnosed or treated for CDI at outside facilities without our knowledge, leading to an underestimation of CDI incidence. Finally, a toxin immunoassay cannot distinguish between new infection and asymptomatic carriage, complicating diagnosis in this population given the similarity of symptoms between CDI and an IBD flare. In fact, the results of these investigations were compromised because the statistical analysis was not limited to participants who had the C. difficile test.

## Conclusions

In conclusion, this study was designed to discover the predictable factors of CDI in IBD patients at King Abdulaziz University Hospital in Jeddah, Saudi Arabia. The results show that IBD patients have a higher morbidity and mortality following a CDI diagnosis, as it leads to an increased rate of hospitalization, colonic involvement, intestinal resections, and enterocutaneous fistula. In regard to medications, there’s a strong relationship between receiving IBD medications and the incidence of CDI. Based on these results, clinicians must consider CDI testing in IBD patients who are on corticosteroids and who are hospitalized to ensure early diagnosis and therapy. Furthermore, future studies are needed to investigate the possibility of predisposition to CDI, particularly among Crohn's patients who are exposed to IBD medication.
